# Investigating ethanol production using the *Zymomonas mobilis* crude extract

**DOI:** 10.1038/s41598-023-28396-4

**Published:** 2023-01-20

**Authors:** Amirhossein Aminian, Ehsan Motamedian

**Affiliations:** Department of Biotechnology, Faculty of Chemical Engineering, Tarbiat Modares University, P.O. Box 14115-143, Tehran, Iran

**Keywords:** Industrial microbiology, Metabolic engineering, Biotechnology

## Abstract

Cell-free systems have become valuable investigating tools for metabolic engineering research due to their easy access to metabolism without the interference of the membrane. Therefore, we applied *Zymomonas mobilis* cell-free system to investigate whether ethanol production is controlled by the genes of the metabolic pathway or is limited by cofactors. Initially, different glucose concentrations were added to the extract to determine the crude extract's capability to produce ethanol. Then, we investigated the genes of the metabolic pathway to find the limiting step in the ethanol production pathway. Next, to identify the bottleneck gene, a systemic approach was applied based on the integration of gene expression data on a cell-free metabolic model. ZMO1696 was determined as the bottleneck gene and an activator for its enzyme was added to the extract to experimentally assess its effect on ethanol production. Then the effect of NAD^+^ addition at the high concentration of glucose (1 M) was evaluated, which indicates no improvement in efficiency. Finally, the imbalance ratio of ADP/ATP was found as the controlling factor by measuring ATP levels in the extract. Furthermore, sodium gluconate as a carbon source was utilized to investigate the expansion of substrate consumption by the extract. 100% of the maximum theoretical yield was obtained at 0.01 M of sodium gluconate while it cannot be consumed by *Z. mobilis*. This research demonstrated the challenges and advantages of using *Z. mobilis* crude extract for overproduction.

## Introduction

Climate change and air pollution are the most challenging global issues, so enormous attention has been investigating the production of biofuels as renewable energy^1,2^. Based on the literature, ethanol is one of the most common biofuels which can replace fossil fuels. Furthermore, ethanol is one of the best alternative fuels among different energy sources for decreasing dependency on fossil fuels. Thus, there is great interest in producing ethanol by using microorganisms like *Saccharomyces cerevisiae*, *Escherichia coli*, and *Z. mobilis*, from renewable biomass instead of relying on petrochemical routes^3–5^. For example, much research has been conducted on improving ethanol production from various substrates such as lignocellulosic and agricultural waste biomass via microbial-based systems, using genetic and computational tools^6–8^. Furthermore, various techniques such as designing culture media, nutrient-feeding strategies, and controlling the pH of the culture media have been applied to gratify the demand to develop industrially competitive microorganisms for producing valuable metabolites^9–12^.

Although microbial-based systems are harnessing in many cases for producing many metabolites and biofuels, there are also some constraints in producing metabolites in microbial-based systems. For example, the efficiency of microorganisms’ processes is often limited by the complexity of microorganism physiology. Complex physiology makes it hard to optimize productivity as there are many regulatory mechanisms in the microorganisms which control pathway flow rates^13,14^. In addition, the viability and life processes of the microorganism spread main resources away from the desired products and lower the final yield. Moreover, products and intermediates can cause constraints such as toxicities in the microorganism. Even the existence of barriers such as membranes in some cases may limit the metabolic engineering approach for regulating pathways^15–17^. For example, one of the main constraints in obtaining higher rates of ethanol production in microorganisms such as *S. cerevisiae* and *E. coli* is that most of the substrates are used for both growth and maintainability. Also, some studies have shown that ethanol decreases the effectiveness of plasma membranes and allows the leakage of essential cofactors and coenzymes^18^. But the main question is how cell-based production systems can overcome these limitations.

The cell-free approach can be a feasible strategy to solve this challenge because it permits bypassing the restrictions of cell growth, membrane barriers, the problems of genetically modified microorganisms, and easy manipulation. The cell-free approach has two methods to build metabolic pathways including in-vitro purified enzymes and crude cell extract^19,20^. For example, Guterl et al.^21^ designed a completely artificial glycolytic reaction cascade in the crude extract of *E. coli* to convert glucose to ethanol. It is comprised of only six enzyme-catalyzed reactions; thus, it needs no phosphorylation requirements. The artificial pathway required only a single cofactor (NAD^+^) and after 19 h, a molar yield of 57% was achieved. Also, cell-free systems can be used to produce a broad variety of molecules, including 2,3-butanediol^22^, terpenes^23,24^, succinic acid^25^, styrene^26^, and n-butanol^27,28^ using purified enzymes or crude cell extracts systems.

This study is divided into two sections. First, we lysed the bacterium *Z. mobilis* with an efficient method to obtain glycolytic pathway enzymes for ethanol production. Then, we investigated whether ethanol production is controlled by the metabolic pathway or is limited by cofactors. So, we added different concentrations of glucose and cofactors to the cell-free reaction environment to start the glycolytic pathway and investigate the capability of this cell-free system for ethanol production. Then, we investigated the genes of the metabolic pathway to find the limiting gene(s) in the ethanol production pathway. In this part, we employed the systems biology approach and an algorithm for the integration of gene expression data with a cell-free metabolic model to identify the limiting step in the glycolytic pathway. Furthermore, The effect of adding NAD^+^ as well as ATP levels and remaining glucose concentration in the *Z. mobilis* crude extract was determined. We further employed gluconate as a new substrate in *Z. mobilis* crude extract, which is known as an intermediate metabolite in the ED pathway and cannot be consumed by the *Z. mobilis* bacteria.

## Materials and methods

### Microorganism and culture media

In this study, the bacterium *Z. mobilis* strain ZM1, known as a high-potential microorganism for ethanol production, was obtained from the Persian-type culture collection. To maintain viability and growth, this strain was periodically cultured on LB agar plates containing agar (15 g/l), peptone water (10 g/l), yeast extract (10 g/l), and glucose (20 g/l). The LB liquid culture containing peptone water (10 g/l), yeast extract (10 g/l), and glucose (20 g/l) was used to prepare seed culture and anaerobic culture. Then, 5 ml of seed culture was inoculated into 250 ml flasks containing 100 ml of fresh LB liquid culture in an incubator at 30 °C and 150 rpm to prepare pre-culture. 5% v/v of pre-culture was inoculated into 1000 ml flasks containing 600 ml of fresh LB liquid culture after 10 h and then became anaerobic using nitrogen gas. This anaerobic culture was used to achieve high cell density and high catalytic power of enzymes in the glycolysis pathway.

### Extract preparation

*Zymomonas mobilis*, containing the glycolytic pathway enzymes for the lysis process, was grown in 1000 ml flasks with 600 ml rich medium (LB) under anaerobic conditions in an incubator at 30 °C. After 10 h, when they reached the mid-exponential phase, they were transferred into six pre-weighted 50 ml Falcon tubes. Then, the contents in the Falcon tubes were harvested by centrifuging at 8000 rpm at 4 °C for 10 min and washed three times with cold Buffer PBS and finally weighed following an established protocol^29^. Buffer PBS contained 8 g/l NaCl, 0.2 g/l KCL, 1.44 g/l Na_2_HPO_4_, and 0.24 g/l KH_2_PO_4_ at 7.4 final pH. All Falcon tubes were kept in an ice-water bath to prevent heat damage to cells.

**Figure 1:**
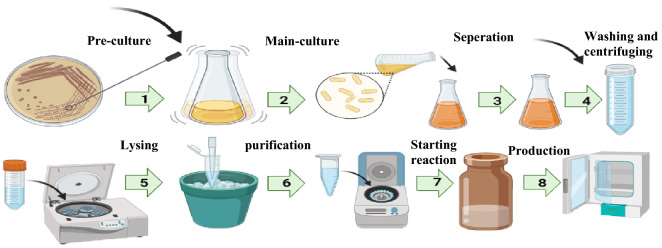
A schematic of bacterial lysis process and cell-free reaction. (1) inoculum from the bacterial plate to the Erlenmeyer flask which contains culture media to prepare pre-culture. (2) inoculum from overnight culture to some Erlenmeyer flask to reach high cell density for the lysis process. (3) transferring Erlenmeyer flask content to some falcon due to separating culture media from bacteria. (4) washing and centrifuging the pellets several times to remove all culture media. (5) lysing bacteria by a sonicator in an ice-water bath to prevent heat damage to bacteria. (6) centrifuging lysis solution to obtain supernatant which contains glycolytic pathway enzymes. (7) transferring bacterial extract to the reaction environment (glass vial) and adding substrate and necessary cofactors to initiate the cell-free reaction. (8) incubation of the glass vial for producing desired product in an incubator.

To generate crude extracts, all pellets were transferred into a marked Falcon tube and re-suspended in 3.5 ml cold Buffer PBS per gram of cell pellet. To lyse cells with the sonication method, the marked Falcon tube was placed in an ice-water bath to prevent heat damage during sonication. A Hielscher sonicator (UP 400S) with a 1.8 mm diameter probe at cycles of 0.6 and 80% of the amplitude was used to lyse cells. To remove cell debris and membranes from the extracted proteins and other metabolites, the crude extract was centrifuged once at 9000 rpm at 4 °C for 15 min. The run-off reaction is not required for further clarification. Next, extracts were transferred into 50 ml penicillin glasses to initiate the glycolytic reaction pathway for ethanol production. The schematic process is presented in Fig. 1.

### Cell-free reaction

Cell-free experiments were carried out at a volume of 2 ml *Z. mobilis* crude extract in 50 ml penicillin glasses in an incubator for 23 h at 30 °C. Penicillin glasses were sealed with paraffin to prevent ethanol leakage from penicillin glasses. To start the ethanol reaction, components in different concentrations according to Table 1 were added to 2 ml *Z. mobilis* crude extract in every experiment. After 23 h, the amount of ethanol produced during the experiment was measured using a GC-2550TG (Teif Gostar Faraz Company, Iran) analyzer. Also, The Glucose concentration was measured enzymatically using a colorimetric glucose oxidase kit (Pars Azmun, Iran). In our experiment, 0.01 M of sodium gluconate was prepared in the cell-free system and placed in an incubator for 23 h to investigate whether the cell-free system based on *Z. mobilis* crude extract could produce ethanol.

**Table 1:** Different concentrations of components for ethanol production in the *Z. mobilis* crude extract.

Experiment number	Glucose (M)	Sodium Gluconate (mM)	NAD^+^ (mM)	EDTA (mM)	MgSO_4_.7H_2_O (mM)
Experiment 1	0.01	–	–	–	0.1
Experiment 2	0.1	–	–	–	0.1
Experiment 3	0.5	–	–	–	0.1
Experiment 4	1	–	–	–	0.1
Experiment 5	–	0.01	–	–	0.1
Experiment 6	0.5	–	–	1	0.1
Experiment 7	0.5	–	–	3	0.1
Experiment 8	1	–	0.01	–	0.1
Experiment 9	1	–	0.2	–	0.1

### Identifying the effective gene and designing the regulatory-defined medium

In this study, the genome-scale metabolic model of *Z. mobilis* ZM1 named iEM439^30^ was used. For constructing in vitro model from a genome-scale metabolic model, all trans-membrane transport reactions (cytoplasm and periplasm/extracellular) were removed from the model as well as extracellular and periplasmic reactions^31^. Since cell-free systems don’t contain membrane compartments, exchange reactions were reassigned to the cytoplasm. The lower and the upper bands of all reversible reactions were bounded to − 1000 and 1000 mmol/gDCW/h respectively, while all irreversible reaction fluxes were limited between 0 and 1000 mmol/gDCW/h. To solve linear programming problems, COBRA and SBML toolboxes, and GLPK (GNU Linear Programming Kit) packages in MATLAB software (R2015b) were applied. Also, the TRFBA algorithm, presented by Motamedian et al.^32^, and expression data^33^ were utilized to find the bottleneck genes controlling ethanol production in *Z. mobilis* crude extract.

In this research, the TRFBA algorithm, which incorporates the metabolic model and expression data, was applied based on Malek’s research^34^ to identify the bottleneck gene(s). After determining the key gene, the BRENDA database was used to investigate regulators of enzymes encoded by this gene. The effect of regulators in different concentrations was experimentally investigated by adding them to the culture medium to enhance the production of ethanol. To evaluate the impact of regulators on ethanol production, a GC-2550TG (Teif Gostar Faraz Company, Iran) analyzer was used to measure ethanol production on every concentration of regulators. All included experiments in this study were repeated four times.

## Results and discussion

### A cell-free platform for ethanol production

**Figure 2:**
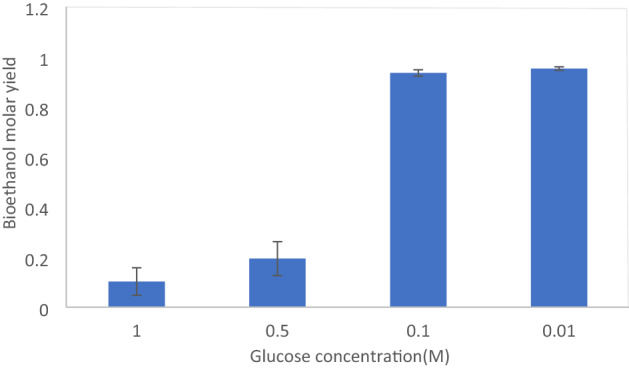
Ethanol molar yield at different glucose concentrations without adding any expensive cofactors in *Z. mobilis* crude extract. Values present averages (n ≥ 3) and error bars present standard deviation.

To initiate the in-vitro reaction and to test whether the cell-free system could produce ethanol with intracellular cofactors, glucose was added to the prepared lysate in the penicillin glasses with different concentrations (0, 0.01, 0.1, 0.5, 1) and then placed into an incubator at 30 °C for 23 h. As shown in Fig. 2, when the concentration of glucose in the crude extract of *Z. mobilis* was zero, the amount of ethanol in the diagram was nearly zero, which means the cell-free system can produce ethanol using crude extract enzymes, and there was no ethanol in the system at the beginning of the process. This experiment was conducted because we hypothesized during a mid-exponential phase, some of the produced ethanol may have remained in the cells and entered the crude extract during the lysis process. Comparing the peaks of the diagram at 0.01 and 0.1 M concentrations of glucose indicates that intracellular cofactors (NAD^+^ and ATP) were carried over from the cytoplasm into the crude extract^35^ and were sufficient for producing ethanol at these glucose concentrations. Although we expected more ethanol production at higher concentrations of glucose (0.5 and 1 M), the result was the same as ethanol production at 0.1 M glucose which means probably, there was an inhibition at high concentrations of glucose in the cell-free system.

So, at low concentrations of glucose (0.01 and 0.1 M), the yield of produced ethanol was nearly 100% of the maximum theoretical yield while at higher concentrations of glucose (0.5 and 1 M), the yield decreased, as shown in Fig. 2. This subject can be because of limitations in metabolic pathways or cofactor shortage that one of the main challenges of cell-free systems is supplying essential cofactors like NAD^+^ and ATP^36^.

Also, glucose concentration was measured in the cell-free system for further studies because we expected the consumed glucose concentration to be proportional to the produced ethanol. It has to be mentioned that glucose concentration was zero in the crude extract before adding glucose. According to Table 2, the cell-free system can consume glucose at 0.01 M glucose concentration and considering this hypothesis that all glucose converted to ethanol due to the adequate amount of essential cofactors during the ED pathway, the maximum theoretical yield was obtained for ethanol. At 0.1 M glucose concentration, the system had the potential to consume all input glucose. Also, at 0.5 and 1 M concentrations of glucose, according to Table 2, the same amount of glucose was consumed because the cell-free system was efficient to consume 0.1 M glucose.

**Table 2:** Bioethanol production and remaining glucose at different glucose concentrations in *Z. mobilis* crude extract.

Initial glucose (M)	Remained glucose (M)	Ethanol yield(*)%
0.01	0.0003 ± 0.00025	100
0.1	0.0006 ± 0.0005	100
0.5	0.396 ± 0.008	20
1	0.895 ± 0.005	10

(*)Ethanol yield is presented as a percentage of the maximum theoretical yield.

In recent research conducted by Dudley et al.^35^ for producing Mevalonate from *E. coli* crude extracts, the effect of NAD^+^ was investigated as the main parameter for high-titer mevalonate production. In another research conducted by Algar and Scopes^37^, the effect of ATP addition in presence of ATPase enzyme in *Z. mobilis* crude extract for producing ethanol was evaluated. However, there is not any experimental report determining the limiting cofactor in the crude extract of *Z. mobilis* for ethanol production. So, to evaluate the cofactor deficiency and find the controlling factor in the crude extract, first, we investigated the genes of the metabolic pathway and applied an activator for its enzyme, then investigated the competition between the NADH/NAD and ATP/ADP ratios for ethanol production.

### Identifying the limiting factor in the *Z. mobilis* crude extract in ethanol production

For further evaluation of the limiting step at high glucose concentration, a new approach was developed to determine the limiting reaction in the pathway of *Z. mobilis* for the conversion of glucose to ethanol. The TRFBA algorithm and *Z. mobilis* metabolic model were applied to find the limiting gene in this section. By calculating shadow price similar to previous works^34^, the ZMO1696 gene was identified as an effective and limiting gene. In previous research, it was investigated that the expression level of the ZMO1696 gene, which is responsible for the synthesis of alcohol dehydrogenase enzyme, plays a critical role in converting aldehydes to alcohol^38,39^.

In the next step, the BRENDA database was used to find a regulator for the enzyme supported by ZMO1696, and finally, EDTA was identified as a regulator at 1- and 3-mM concentrations^40^. So, EDTA was added to the crude extract and no significant increase in ethanol production was observed at high glucose concentrations (0.5 and 1 M). In conclusion, according to Yang et al.^39^ research, the alcohol dehydrogenases had high activity in the *Z. mobilis* crude extract, although the ZMO1696 gene had a low expression. Therefore, these results indicate that the activity of enzymes is not limiting step at high glucose concentrations and hence, the lack of essential cofactors can still be considered as the main bottleneck.

### Evaluation of cofactors’ effect on ethanol production

In this experiment, we investigated the effects of NAD^+^ cofactor in ethanol production because it plays a crucial role in cell-free systems^41,42^. First, we assumed that if the bottleneck is downstream of the ED pathway, the balance ratio of NAD^+^/NADH faces a problem, and converting NAD^+^ to NADH will be stopped because of the NAD^+^ shortage. To evaluate this hypothesis, NAD^+^ at 0.01 and 0.2 mM concentrations were added to the cell-free system. The result revealed no increase in ethanol yield at 1 M glucose concentration and suggested that NAD^+^ is not a limiting agent at high glucose concentrations.

**Figure 3:**
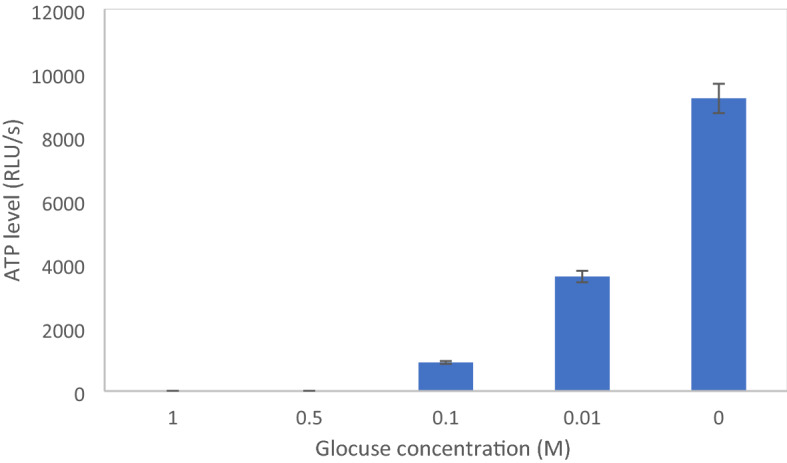
ATP levels after 23 h of incubation for ethanol production at different glucose concentrations in *Z. mobilis* crude extract. Values represent averages (n ≥ 3) and error bars represent 1 s.d.

Considering NAD^+^ is not the bottleneck of the ethanol production pathway, the effect of the other important cofactor ATP was evaluated. In fact, glucose cannot be converted to glucose 6-phosphate if there is a lack of ATP at high glucose concentrations, and hence, the ED pathway stops. in this part, the ATP levels for different glucose concentrations (0, 0.01, 0.1, 0.5, and 1 M) at the end of the process were measured as shown in Fig. 3. When glucose concentration was zero in the cell-free system, the amount of ATP cofactors was the highest because of no metabolic activity. In the low glucose concentration (0.01 and 0.1 M), the amount of initial and produced ATP cofactor in the system was sufficient to convert all glucose into the product. Although, with the increase in glucose concentration (0.1 M), the amount of ATP cofactor drastically decreased. As glucose concentrations increased to 0.5 and 1 M in the cell-free system, the amount of ATP was not enough to supply energy for converting all the glucose to ethanol. Thus, the results indicate that the amount of ATP and imbalance of ADP/ATP ratio is limiting in cell-free systems for the consumption of glucose at high concentrations. Similar to our result, Welch ans Scopes^43^ experimentally demonstrated that the inconsistency of the ADP/ATP ratio was the main bottleneck in the glycolytic pathway of the *S. cerevisiae* crude extract for ethanol production.

### Using sodium gluconate as an alternative carbon source

Sodium gluconate was selected as an alternative substrate because firstly, *Z. mobilis* cannot grow on it as the only carbon source^44,45^ and hence, production in the crude extract demonstrates an advantage of cell-free systems, and secondly, it can be converted to ethanol in the *Z. mobilis* crude extract without adding any extra cofactors. This substrate has some attractive characteristics: (1) it can be metabolized only using the NADH cofactor and does not require ATP cofactor for starting the ED pathway; (2) it is an intermediate of the ED pathway involving fewer steps for producing ethanol. In the present study, we observed the high conversion of sodium gluconate (95% ± 4% of maximum theoretical yield) at 0.01 M to ethanol after 23 h of incubation of *Z. mobilis* crude extract with sodium gluconate as a new substrate.

## Conclusions

In this work, we showed *Z.mobilis* crude extract's capability to produce ethanol via the ED pathway without applying any expensive cofactors (ATP and NAD^+^) at low glucose concentrations (0.01 and 0.1 M). We also investigate whether ethanol production is controlled by the genes of the metabolic pathway of ethanol production or is limited by cofactors. To study the metabolic pathway, the transcriptomic data with a cell-free metabolic model was integrated. ZMO1696, was determined as the bottleneck gene and an activator for its enzyme was added to the extract but the result revealed no increase in bioethanol yield. In continuation, the effects of NAD^+^ and ATP were investigated. Finally, by measuring ATP levels in different glucose concentrations, we found that the ATP cofactor was a limiting agent in the Z. mobilis crude extract. Furthermore, to the best of our knowledge, this research is the first demonstration of producing ethanol using gluconate as a carbon source in the *Z. mobilis* crude extract. In contrast, the *Z. mobilis* wild type cannot metabolize gluconate as a carbon source via the ED pathway.

In summary, our research shows the high activity of enzymes in *Z. mobilis* crude extract as we obtained the maximum theoretical yield of ethanol production. It also proves that our system can compete in price and outcome with cellular-based systems, and ATP addition to the crude extract can be beneficial.

## Data Availability

All generated or analyzed data during this study are included in this paper.
